# Supplementary material for: A cell-level discriminative neural network model for diagnosis of blood cancers
Source: Bioinformatics. 2023 Sep 26;39(10):btad585. doi: 10.1093/bioinformatics/btad585 (PMC10563151; doi:10.1093/bioinformatics/btad585)
Supplement: btad585_Supplementary_Data [file btad585_supplementary_data.zip › Appendix.pdf]

A Appendix

A.1 Specific hyperparameters

For CSNN-logistic, the tuning hyperparameters used were the learning rates (10^{-3}, 10^{-4}, 10^{-5}, 10^{-6}), the w parameter (0.75, 0.85, 0.90, 0.95, 0.99, 0.995), and also an architecture search, where we test two kinds of architectures for the s function, one with ℓ dense layers (1, 2, 3), and of size n (3, 6, 10, 15). The other kind of architecture is a funnel that starts at size 2^n (for $n = 8, 4, 2$), half at each step and ends at size 1.

For CSNN-reg, the hyperparameters tuned were the learning rate (10^{-3}, 10^{-4}, 10^{-5}, 10^{-6}), negative penalty λ (1, 5, 15, 30), and the same architecture search as the logistic method for s.

For DeepCellCNN, we use the following hyperparameters: the learning rate (10^{-3}, 10^{-4}, 10^{-5}, 10^{-6}), and then varying the architecture, with an option for the hidden layers (3, 6, 10, 1), the embedding dimension size (3, 6, 10, 15), and the hidden layer of the head of the neural network (3, 6, 10, 15).

For CellCNN we use the following hyperparameters: the learning rate (10^{-3}, 10^{-4}, 10^{-5}, 10^{-6}), then the architecture, with the number of filters (3, 6, 10 and 15), and the dropout (from 0.1 to 0.9 with a step every 0.1).

A.2 Interpretation of cell-level scores as gates

Traditional manual gating methods use a similar strategy to the method we just described: humans look at 2D plots of marker values and draw a box where they believe pathogenic cells are located for that pair of markers. This is repeated using additional 2D plots, filtering out the cells outside of the bounding box at each step. In the end, human gaters end up generating a similar function s', where

$$s'(x;G) = \begin{cases} 1 & \text{if } x \in G \\ 0 & \text{otherwise} \end{cases}, \tag{1}$$

where G is a set that describes the space after all the filtering done by a human is applied. We can interpret the score of a cell $s_{i,j} = s(x_{i,j}, \phi)$ as the probability of it being a pathogenic cell. This is because when using L_{MSE} and L_{LL} as our mappings when scoring a whole sample, the final probability is proportional to the mean of the probabilities in the sample. If we consider the set G such that

$$G = \{x | s(x, \phi) > 0.5\}, \tag{2}$$

The function s represents the human filtering done through s'. G can also be interpreted as the location in marker space where pathogenic cells are likely to be found, since it is the region where s has a high activation, and high activation is proportional to a high probability score. These scores can also be any number between 0 and 1, representing the probability that a cell is pathogenic. In practice, these are usually close enough to 0 or 1, to the point where they're all essentially non-pathogenic or pathogenic.

It is worth noting that s has more representation power than s', this is because while s is represented by a cell scoring neural network, which allows it to have any arbitrary shape, s' is represented by a hierarchy of 2D boxes, which can only be reduced to n dimensional boxes. This means that, if we consider the case of two markers A and B, where cancer cells are both A^-B^- and A^+B^+ and healthy cells A^+B^- and A^-B^+, a single 2D box cannot filter cancer cells based on these markers, whereas a neural network has no trouble making these distinctions.

A.3 Ablation study of the method

Three ablation tests were conducted to determine if the CSNN methods are improved by either the density difference initialization or the post initialization fine-tuning. The first test consisted of running the initialization score as a classification score without involving any neural network - denoted as Init-only. The other two ablation tests used CSNN-Reg and CSNN-Class without the density difference initialization - denoted as CSNN-Reg-noinit and CSNN-Class-noinit. The tests were run for 5 times and the best scores are reported in Table 1. These results indicate that both the initialization and the post-initialization fine-tuning have a significant impact on the accuracy of the models.

A.4 Clustering-based independent analysis

We designed an ad hoc data clustering application to identify the different clusters of cells between the non-cancer and cancer samples. A simple K-means clustering was applied to the original data space without any "black box" feature transformation or preprocessing steps. Due to the long runtime of K-means on large datasets, we selected and merged a small number of samples to create a template for identifying the optimal positions of the cluster centroids using 300 K-means iterations. Then we applied these centroids as initial seeds to generate final clustering results for the other samples, using only two K-means iterations. K=100 (i.e., 100 clusters) was used to overpartition the data so that both abundant and rare cell populations can be identified for being compared between the cancer and non-cancer cohorts. The clusters that were found in the cancer cohort only will be output as "pathologic cells". Specifically, we selected 10 CLL positive samples with >50% tumor burden and 10 CLL negative samples from the training set to create the CLL template. As the B-ALL files are much larger than the CLL, only three B-ALL positive samples with 90% or larger tumor burden were pooled to create the B-ALL template.

We then used the nonparametric Wilcoxon Rank Sum test to select the clusters that are significantly amplified in the cancer cohort with a p-value cut off = 0.05. These clusters are the "global cancer clusters" identified at the cohort-level, i.e., they are seen in most of the cancer samples. However, it is known that the B-ALL has much larger phenotypical heterogeneity than CLL, which requires us to create an additional rule to identify the sample-specific B-ALL blasts. We defined an empirical rule based on our observation: if the proportion of a cluster in a testing sample is larger than the average proportion + 3 * standard deviation of the same cluster in the healthy samples of the training set, this cluster is regarded as a cancer cell cluster for this testing sample, no matter this cluster is found in other cancer samples or not. Finally, both the global and the sample-specific cancer cell clusters were output as the predicted cancer cells for the B-ALL samples.

A.5 Initializing a CSNN Model using Density Estimates

We can improve the quality of the CSNN models by using information related to the densities in marker space to initialize the models. We begin by generating initial estimates of cell-level scores $s'_{i,j}$ for each cell j in each sample i in the training data. Consider first the case of real-valued y_i targets (i.e., sample burdens). For samples with $y_i = 0$, all cell-level scores are zero by definition, i.e., $s'_{i,j} = 0$, assuming that all cells in healthy samples are non-pathogenic. For samples with a disease diagnosis ($y_i > 0$) we use the following approach:

$$\begin{aligned} s'_{i,j} &= P(z_{i,j} = 1 | x_{i,j}, y_i > 0) \\ &= 1 - P(z_{i,j} = 0 | x_{i,j}, y_i > 0) \\ &= 1 - \frac{P(x_{i,j} | z_{i,j} = 0, y_i > 0) P(z_{i,j} = 0 | y_i > 0)}{P(x_{i,j} | y_i > 0)} \end{aligned}$$

where the last expression follow from Bayes rule. We can estimate each of the three terms on the right hand side from the training data as follows. The term in the denominator, $P(x_{i,j}|y_i > 0)$, is the marginal probability density function (PDF) of marker measurements for sample i, given that sample i has a disease diagnosis: this PDF can be estimated straightforwardly using kernel density estimation (KDE). The first term in the numerator, $P(x_{i,j}|z_{i,j} = 0, y_i > 0)$ is the probability density for non-pathogenic cells in a sample with a disease diagnosis. We can approximate this by assuming that $P(x_{i,j}|z_{i,j} = 0, y_i > 0) = P(x_{i,j}|z_{i,j} = 0) = P(x_{i,j}|z_{i,j} = 0, y_i = 0)$, i.e., that the PDF of non-pathogenic cells in marker space is the same in both positive and negative samples. We further assume that the density $P(x_{i,j}|z_{i,j} = 0, y_i = 0)$ does not vary from sample to sample, allowing us to pool all $x_{i,j}$ measurements from all the negative samples (which have $y_i = 0$ and $z_{i,j} = 0$ by definition) and again use KDE to estimate this density. The second term in the numerator, $P(z_{i,j} = 0|y_i > 0)$, is equal to $1 - y_i$, under

Table 1. Area under the ROC for each ablation and result.

	B-ALL	CLL
CSNN-Reg-noinit	78.1%	91.2%
CSNN-Reg	79.1%	94.4%
CSNN-Class-noinit	76.3%	78.0%
CSNN-Class	77.6%	76.3%
Init-only	72.8%	79.3%

the assumption that y_i (the sample burden) corresponds to the fraction of cells in sample i that are pathogenic. For the situation where the training data only contains binary labels $y_{i,j} \in \{0, 1\}$, i.e., the CSNN-Class model, we again estimate scores $s'_{i,j}$ via Bayes rule, but using additional approximations for each of the three required terms in the absence of burdens y_i.

Finally, given scores $s'_{i,j}$ from the density-based approach above (for all cells for all samples in the training dataset), a feedforward neural network, parametrized by weights ϕ', is trained to create an initial cell-level model that can approximate the density-based scores via the neural network. The trained weights ϕ' of this neural network are then used to initialize the weights in training of an end-to-end CSNN network (CSNN-Reg or CSNN-Class, using L_{MSE} and L_{LL} respectively as described earlier). We found in practice that this density-based initialization significantly improves the quality of the final sample-level disease predictions. Full details on KDE methods, approximations for binary y_i labels, and training of the initial network, are provided in the Supplement.

Note that one could in principle use the density-based approach alone to build a sample-level prediction model, by using the density-based models from the training to generate scores $s'_{i,j}$ per cell for a new sample. A prediction for y_i could then be based for example on thresholding the sum of cell-level scores $\sum_j s'_{i,j}$. However, a purely density-based approach may be sensitive to modeling assumptions, whereas the sample-level discriminative training of the CSNN can allow the model to further tune the initial parameters ϕ' to produce scores $s_{i,j}$ that are directly optimized for robust prediction of burdens or likelihood of disease.

A.6 Comparison of results of CSNN with other approaches

2D dot plots for visually comparing CSNN with CellCNN and DeepCellCNN across all CLL and B-ALL samples can be found at GitHub:

https://github.com/JCVenterInstitute/DAFi-gating/tree/master/CSNN/Comparison_with_CellCNN_DeepCellCNN.
