# Supplementary figures and images for: A cell-level discriminative neural network model for diagnosis of blood cancers
Source: Bioinformatics. 2023 Sep 26;39(10):btad585. doi: 10.1093/bioinformatics/btad585 (PMC10563151; doi:10.1093/bioinformatics/btad585)

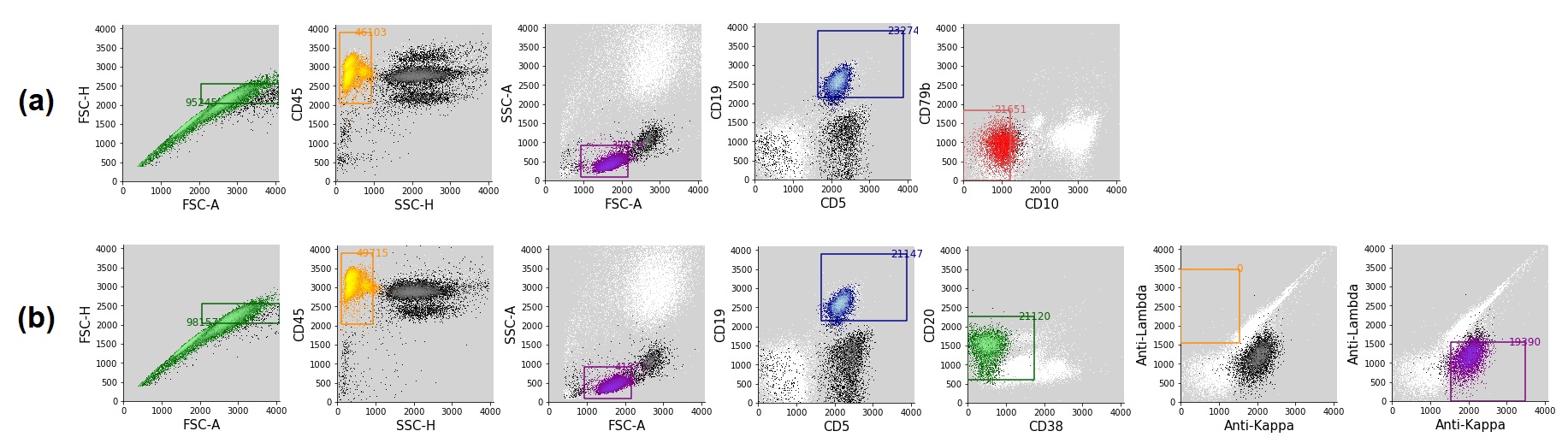

Supplement: btad585_Supplementary_Data [file btad585_supplementary_data.zip › Supplemenatry_Figure1.jpg]

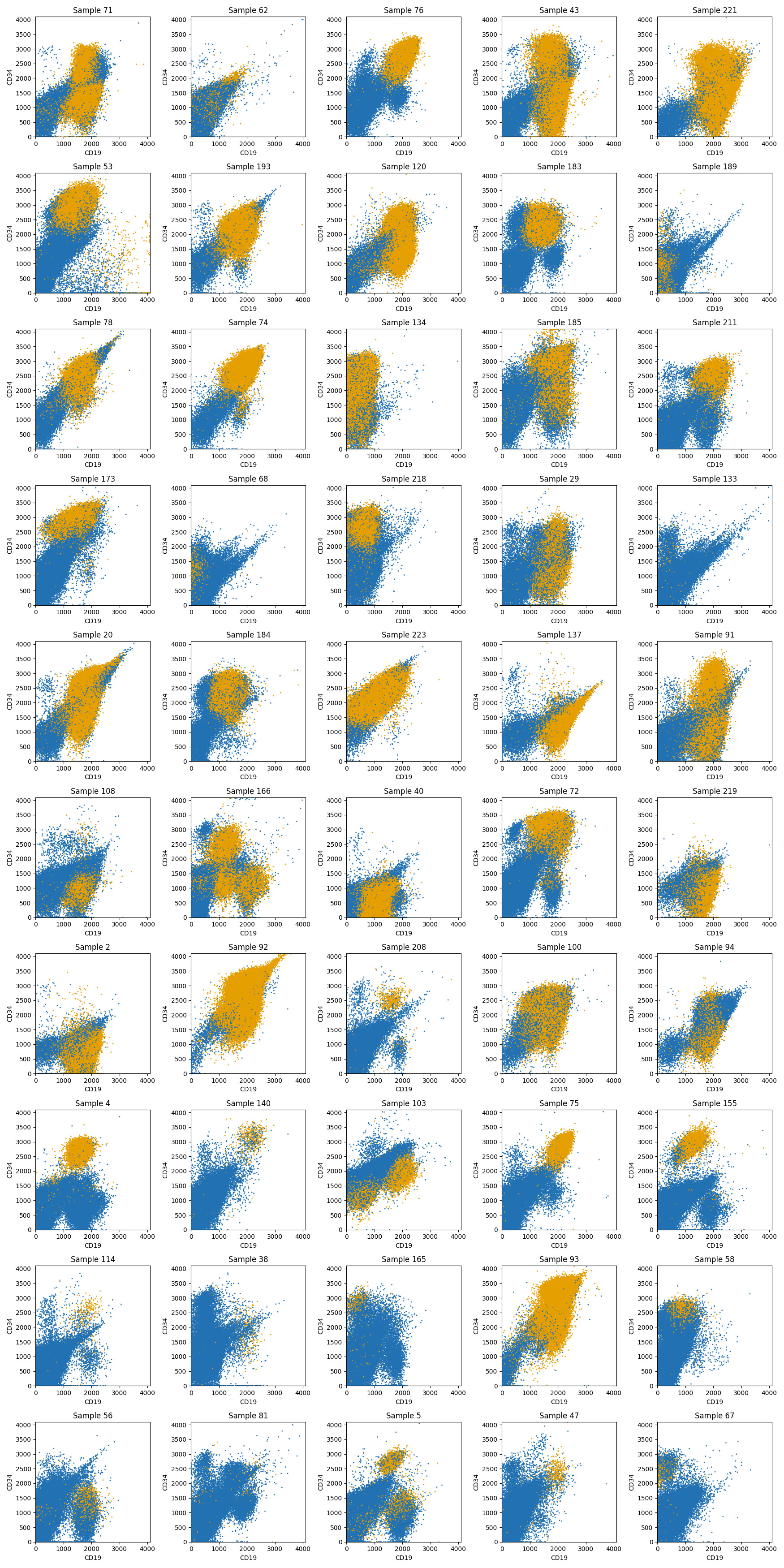

Supplement: btad585_Supplementary_Data [file btad585_supplementary_data.zip › Supplementary_Figure3.png]

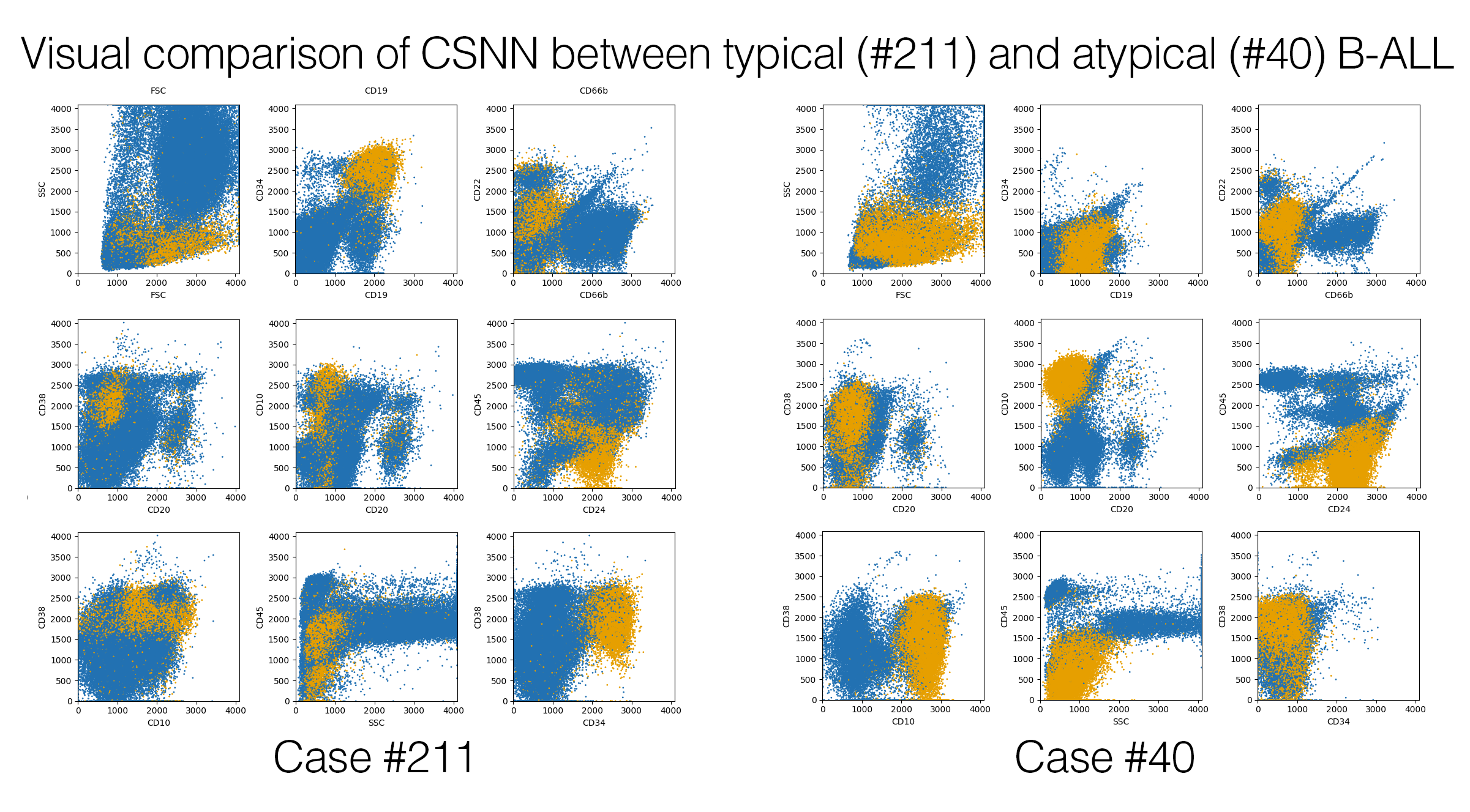

Supplement: btad585_Supplementary_Data [file btad585_supplementary_data.zip › Supplementary_Figure5.png]

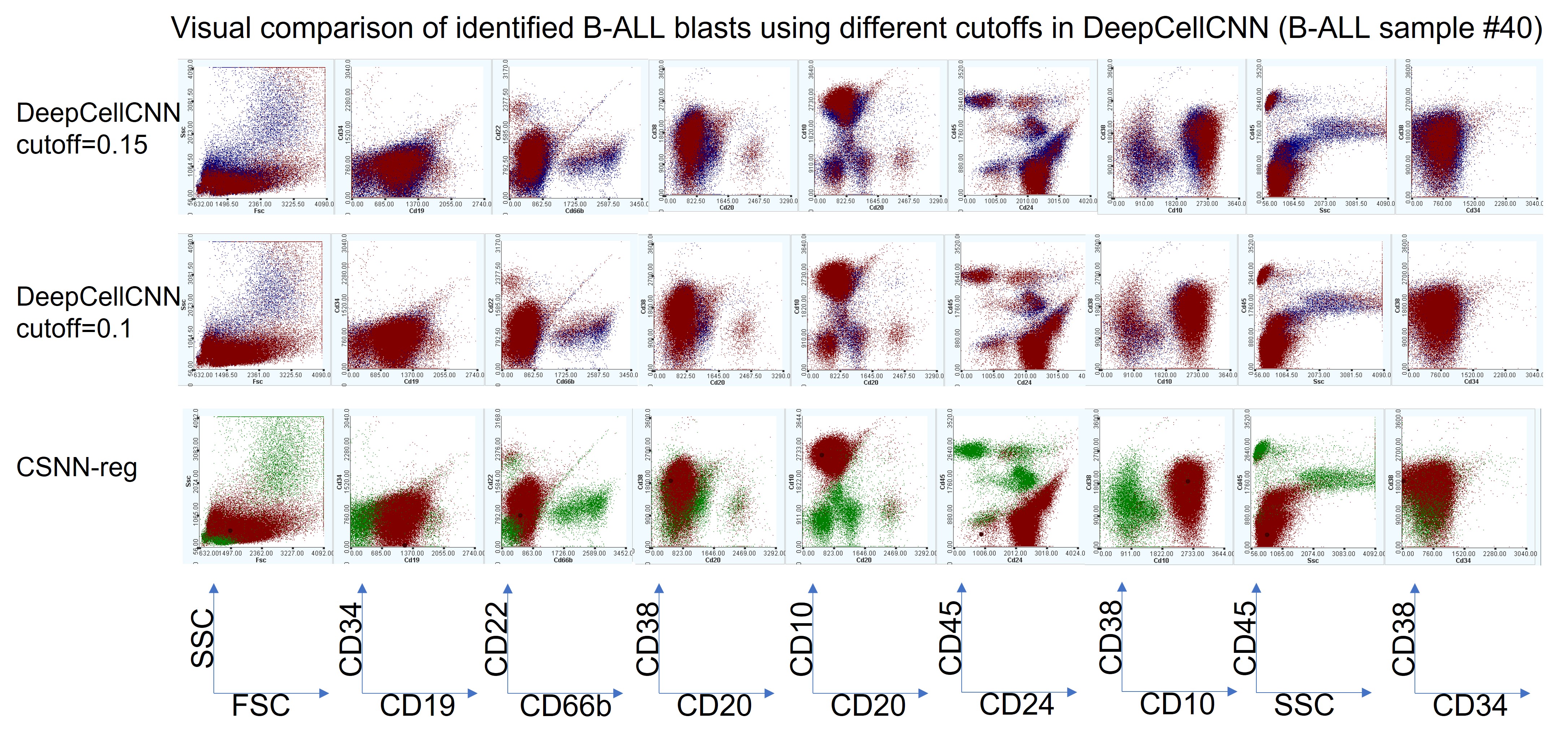

Supplement: btad585_Supplementary_Data [file btad585_supplementary_data.zip › Supplementary_Figure_6.jpg]

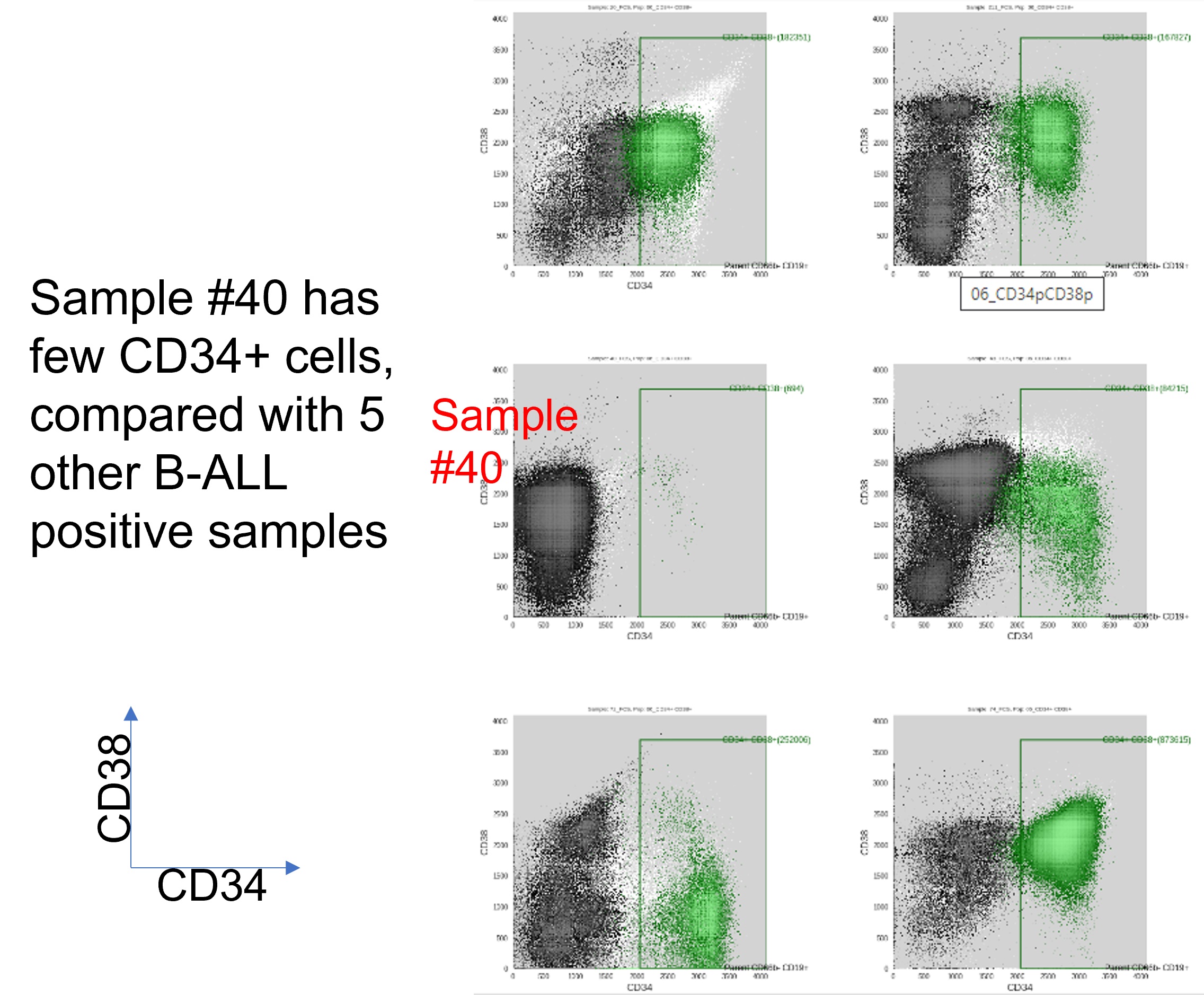

Supplement: btad585_Supplementary_Data [file btad585_supplementary_data.zip › Supplementary_Figure4.jpg]

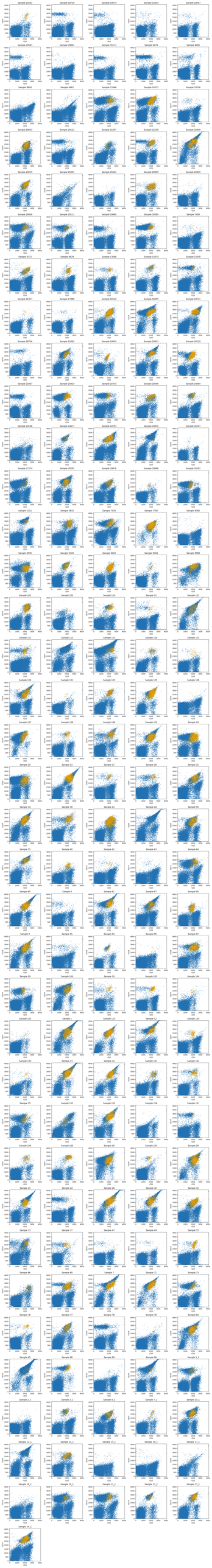

Supplement: btad585_Supplementary_Data [file btad585_supplementary_data.zip › Supplementary_Figure2.png]
